# 0994. Development of ventilatory-induced lung injury depends on energy dissipated into respiratory system

**DOI:** 10.1186/2197-425X-2-S1-P79

**Published:** 2014-09-26

**Authors:** M Gotti, C Chiurazzi, M Amini, C Rovati, M Brioni, A Cammaroto, S Luoni, C Bacile di Castiglione, G Rossignoli, C Montaruli, K Nikolla, M Monti, D Dondossola, I Algieri, T Langer, M Cressoni, L Gattinoni

**Affiliations:** Fisiopatologia Medico-Chirurgica e dei Trapianti, Università degli Studi di Milano, Milano, Italy

## Introduction

Mechanical ventilation with high volumes/pressures induces ventilatory induced lung injury (VILI). During each breath, part of the energy transmitted from ventilator to respiratory system is given back as elastic recoil and part is dissipated into the respiratory system; this amount of energy is measured by the hysteresis area of the pressure-volume (PV) curve of the respiratory system. Total dissipated energy into respiratory system, or dissipated inspiratory potency, equals to energy dissipated during every single breath multiplied by the respiratory rate (RR).

## Objectives

To measure dissipated inspiratory potency throughout lung parenchyma and to asses if the reduction of the energy load into the respiratory system, obtained reducing RR, delays the development of VILI.

## Methods

Ten piglets, after general anaesthesia induction, were ventilated at lethal tidal volume (TV), defined on strain > 2.5 (which is known to cause lung oedema within 54 hours), but at different RR: 4 pigs at RR 3, 3 pigs at RR 6, 1 pig at RR 9 and 2 pigs at RR 15. We measured PaO_2_/FiO_2_ ratio, plateau pressure, and PV curve hysteresis in dynamic and static conditions at the beginning and at the end of the experiment and lung weight at the end of experiment.

## Results

**Figure 1:**
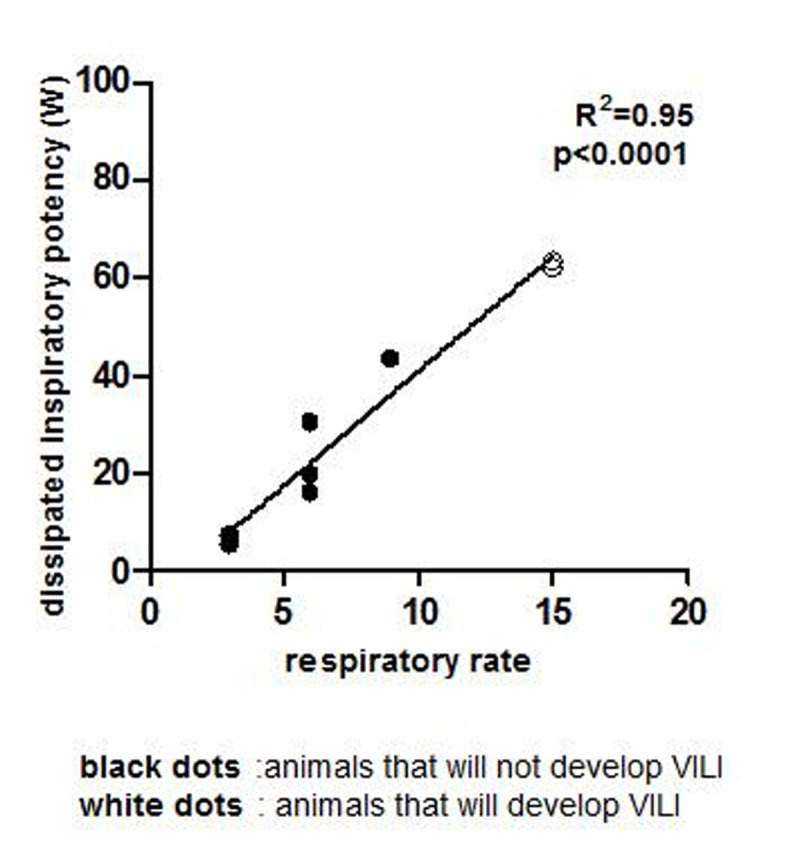
Figure 1

Inspiratory potency set at the beginning of the experiment had a linear relationship with RR (R^2^=0.95, p< 0.001) (Fig.1).

There were no differences in baseline conditions and in TV setting between groups. Piglets ventilated at RR 15 developed lung oedema (final lung weight: 550±28 g vs 287±49 g, p< 0.001). PaO_2_/FiO_2_ ratio and plateau pressure at the end of the experiment were different in piglets ventilated at RR15 (177±9 mmHg vs 437±77 mmHg, p< 0.01; 33±3 cmH_2_O vs 22±5 cmH_2_O, p< 0.05, respectively), as compared to piglets ventilated at RR3, 6 and 9. Energy dissipated into the respiratory system increased only in pigs who developed lung oedema, from 63±1 W at the beginning to 110±28 W at the end of the experiment.

## Conclusions

The development of VILI (lung oedema) depends on inspiratory potency dissipated into the respiratory system.

